# Effects of Adhesive Coating on the Hygrothermal Aging Performance of Pultruded CFRP Plates

**DOI:** 10.3390/polym12020491

**Published:** 2020-02-23

**Authors:** Xinkai Hao, Guijun Xian, Xiangyu Huang, Meiyin Xin, Haijuan Shen

**Affiliations:** 1 Key Lab of Structures Dynamic Behavior and Control of the Ministry of Education, Harbin Institute of Technology, Harbin 150090, China; xinkai.hao@adelaide.edu.au; 2 Key Lab of Smart Prevention and Mitigation of Civil Engineering Disasters of the Ministry of Industry and Information Technology, Harbin Institute of Technology, Harbin 150090, China; 3 School of Civil Engineering, Harbin Institute of Technology, Harbin 150090, China; 4 Acrylic Fiber Division, Sinopec Shanghai Petrochemical Co., Ltd., Shanghai 200540, China; huangxy.shsh@sinopec.com (X.H.); xinmy.shsh@sinopec.com (M.X.); shenhj.shsh@sinopec.com (H.S.)

**Keywords:** CFRP pultruded plates, adhesive coating, water immersion, mechanical behavior

## Abstract

Bonding of carbon fiber reinforced polymer (CFRP) plates to a concrete member is a widely used strengthening method. CFRP plates used in construction degrade due to harsh environmental conditions such as high temperature or alkaline solution seepage from concrete. However, the adhesive between CFRP plates and concrete may have a positive effect on the durability performance of CFRP plates. In this paper, the long-term performance of both naked and adhesive coated CFRP pultruded plates subjected to different-temperature water or alkaline solution (20, 40 and 60 °C) are investigated to evaluate the protective effect of adhesive on CFRP plates. It is found that the adhesive coating can slow the deterioration of mechanical properties especially the tensile properties and fiber-matrix interfacial properties. The water absorption mechanism of CFRP plates was also investigated.

## 1. Introduction

Fiber reinforced polymers are accepted as construction materials due to their outstanding performance and resistance to corrosion compared with concrete and steel [1]. Moreover, fiber reinforced polymer plates have been widely used as strengthening materials of concrete members [2,3,4,5]. The long-term performance of FRP materials in harsh environments such as hygrothermal or alkaline environments is significant for their application [1] and has been widely investigated [6,7,8,9,10].

It is believed that the water uptake of FRP composites plays a key role in the degradation of their mechanical and thermal properties, especially in water [11,12], alkaline solution (simulation of concrete pore solution) [6,13] and salt solutions (simulation of seawater) [14,15]. Ficker’s model [16] and a two-stage model [17] were proposed to simulate the water-uptake behavior of FRP composites [13,18,19,20]. Finite element analysis with the aid of ABAQUS was also conducted to investigate the water-uptake process based on Ficker’s model [21,22]. These approaches assume the constant diffusion parameter *D*, which will be further investigated in this paper.

Water diffusion in FRP composites in a hygrothermal environment may cause the swelling of the FRP materials [23] and consequently localized strain and stress and subsequently the degradation of the bonding between the fiber and resin matrix [8,10]. This may seriously affect the mechanical performance of FRP composites [10]. It is also found that hygrothermal exposure may cause the swelling of the matrix and if the matrix experiences an excessively harsh environment such as 80 °C water, the matrix may lose weight and subsequently the suffer mechanical properties damage [9]. However, in simulated concrete pore solution environments, the adhesive between the concrete and FRP may have a positive effect on the durability of FRP materials, which is normally ignored.

In this paper, the positive effect of adhesive on the durability of pultruded CFRP composites under both water immersion and simulated concrete pore alkaline solution conditions at different temperatures is investigated. The corresponding thermal and mechanical properties, including tensile properties and in-plane shear strength were tested for specimens under different aging conditions to understand the degradation mechanism and evaluate the usability of FRP which is under concrete pore alkaline solution.

## 2. Experimental Program

### 2.1. Materials and Sample Preparation

The pultruded CFRP plates were made from epoxy resin and T-300 grade carbon fiber and were unidirectional. The resin matrix is bisphenol-A diglycidyl ether (DGEBA) and the hardener is a methyl tetrahydrophthalic anhydride (MeTHPA). The tensile strength and elastic modulus of the CFRP plates are 1.85 GPa and 173 GPa respectively. The fiber volume fraction (*V_f_*) equals approximately 65.7%.

**Figure 1:**
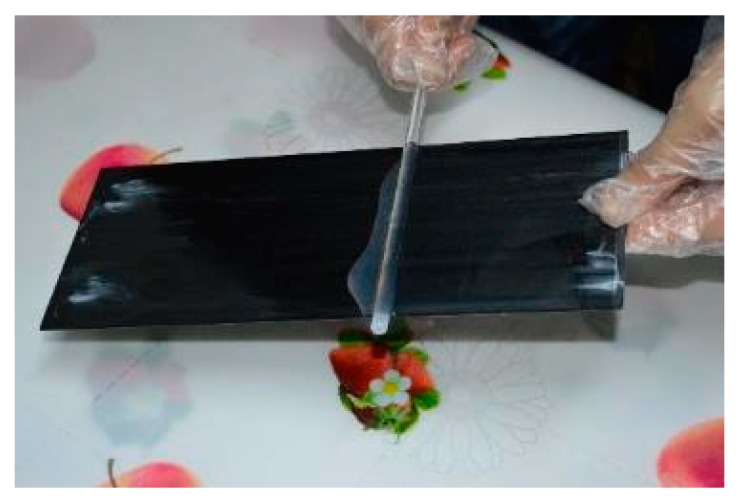
Coating CFRP plate.

**Figure 2:**
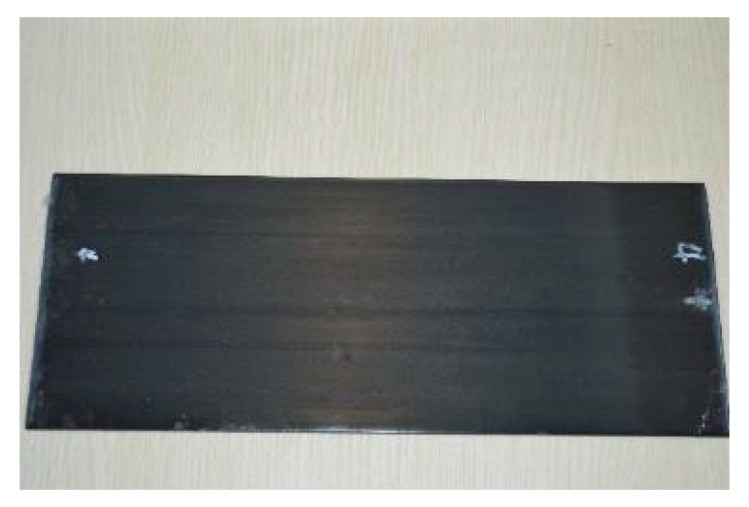
Adhesive coated CFRP plate.

CFRP plates were to a size of 250 mm × 100 mm × 1.22 mm in the fiber direction. Half of the specimens were coated by T1 construction adhesive (the main component is an epoxy resin) and the procedure is summarized as follows: the adhesive was smeared uniformly by using the glass bar as shown in Figure 1; then the plate was cured in laboratory oven for twelve hours; in the end, coated CFRP plates with the total thickness equal to around 1.44 mm were chosen for the tests and one example is shown in Figure 2.

### 2.2. Immersion Media

**Figure 3:**
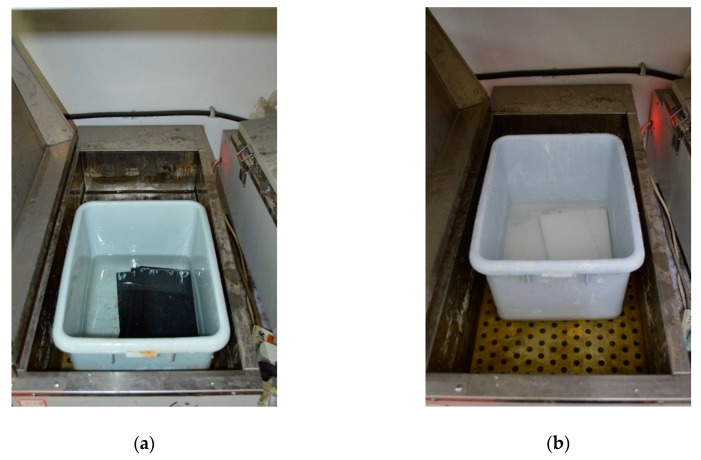
Specimens immersed in: (**a**) Water; (**b**) Alkaline solution.

Both the coated and naked CFRP plates were immersed in either distilled water or simulated alkaline solution with the components listed in Table 1. This alkaline solution is prepared according to the guide test methods proposed by American Concrete Institute [24]. The water and alkaline solution immersion environments are shown in Figure 3a,b, respectively. The immersion temperatures were set as 20, 40 and 60 °C. The immersed plates were separated by a 250 mm × 15 mm × 1.22 mm plate to ensure sufficient contact between the specimens and their immersion environment.

### 2.3. Test Methods

#### 2.3.1. Water Uptake

Both naked and coated CFRP plate specimens immersed in the water or alkaline solution were weighed to quantify the water gain which can be written as:

(1) $$M_{t}=\frac{W_{t}-W_{0}}{W_{0}}\times 100\%$$

where *W_0_* is the initial weight of the samples and *W_t_* is the mass of the aging samples.

#### 2.3.2. Thermogravimetric Analysis

The samples immersed in water for 135 days and the initial samples before immersion were measured with a simultaneous thermal analyzer (STA-449 C, NETZSCH, Selb, Germany). The temperature ranged from 0 °C to 800 °C at a rate of 10 °C/min under the nitrogen environment. The coated CFRP plates after removing adhesive and naked plates were tested and the weight of samples was around 5 mg.

#### 2.3.3. Tensile Tests

The tensile properties of the CFRP plates were tested according to ASTM D3039/D3039M [25] using a universal mechanical testing machine (WDW-100D, Jinan Shijin, Jinan, China). The tensile tests were performed in the fiber direction. The size of the testing samples is 250 mm × 15 mm × 1.22 mm. Five specimens were tested for each immersion temperature and environment, and the average results were recorded.

#### 2.3.4. In-Plane Shear Strength Test

**Figure 4:**
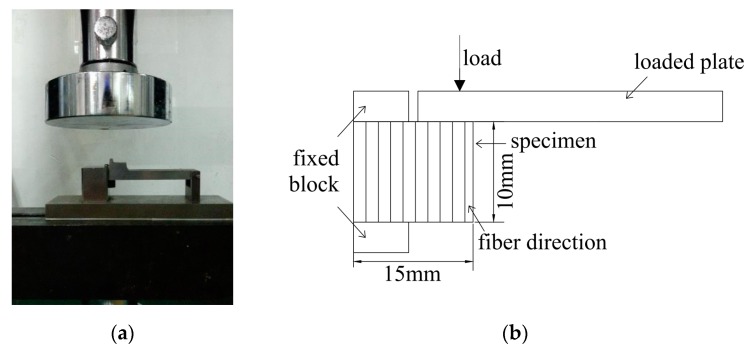
In-plane shear test: (**a**) Realistic diagram; (**b**) Schematic diagram.

The in-plane shear strength test is shown in Figure 4a and the schematic graph is shown in Figure 4b. The specimen size for this test is 15 mm × 10 mm × 1.22 mm and the fiber direction, height and width of the specimen is shown in Figure 4b. Five specimens were tested for each immersion temperature and environment to record the average strength. Failure load *P* is resisted by the shear strength *τ* along the failure plane which can be written as:

(2) $$\tau =\frac{P}{\mathrm{hb}}$$

where *h* and *b* are the height and thickness of the sample cross-section.

#### 2.3.5. Fourier Transform Infrared Test

The initial naked CFRP plates and those immersed in water or alkaline solution for three months at either 20 °C or 60 °C were tested by a FTIR 783 Spectrophotometer (Perkin Elmer, Waltham, MA, USA). The tablet made by one to two mg immersed sample and 200 mg potassium bromide was tested within the wave-number range from 400 cm^−1^ to 4000 cm^−1^.

#### 2.3.6. DMTA Test

Dynamic mechanical thermal analysis (DMTA) was conducted on a Q800 system (TA Instruments, New Castle, DE, USA) using the single cantilever method under a frequency of 20 Hz. The specimen size is 30 mm × 8 mm × 1.22 mm and the temperature ranged from 25 °C to 200 °C at a rate of 5 °C/min.

## 3. Results and Discussion

**Figure 5:**
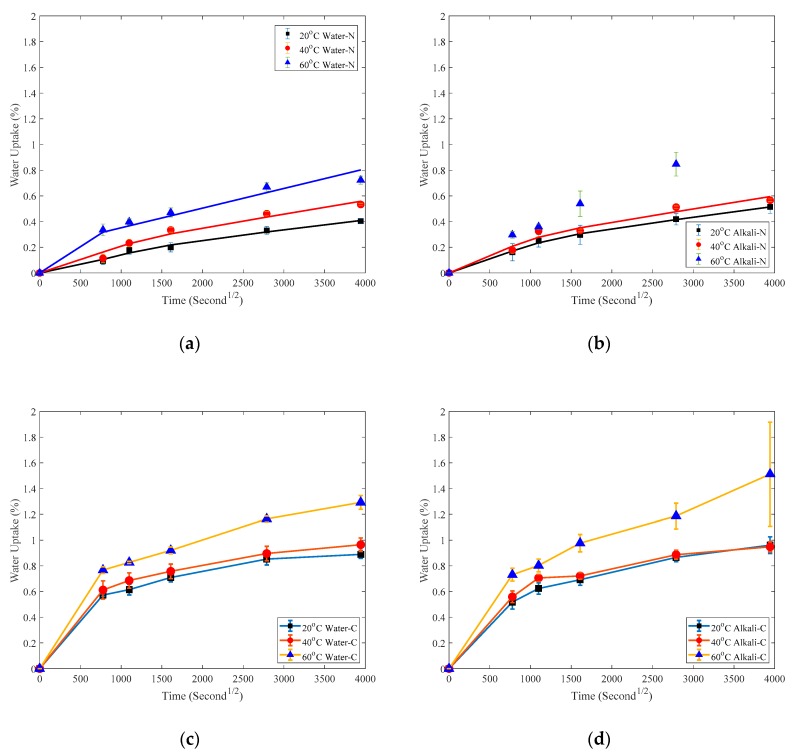
Water uptake of: (**a**) Naked plate immersed in water; (**b**) Naked plate immersed in alkaline solution; (**c**) Coated plate immersed in water; (**d**) Coated plate immersed in alkaline solution. In Figure 5a,b the solid lines represent the two-stage model curve fittings.

In this section, the water uptake behavior is described first and then the mechanical properties and chemical analysis. It is worth noting that the samples are labelled in the figure legends in this section by adding the aging condition and the coating status. For instance, 20 °C Water-N in Figure 5a and 20 °C Alkali-C in Figure 5d stand for the naked specimen immersed in 20 °C water and coated specimen immersed in 20 °C alkaline solution, respectively.

### 3.1. Water Uptake

Water uptake of specimens can be quantified from Equation (1) and the water uptake of both naked and coated CFRP plates is shown in Figure 5. In Figure 5a,b, the water uptake is fitted by the following two-stage water uptake model [17]:

(3) $$M_{t}=M_{\infty }\left(1+k\sqrt{t}\right)\left\{1-\exp\left[-7.3{\left(\frac{\mathrm{Dt}}{h^{2}}\right)}^{0.75}\right]\right\}$$

**Figure 6:**
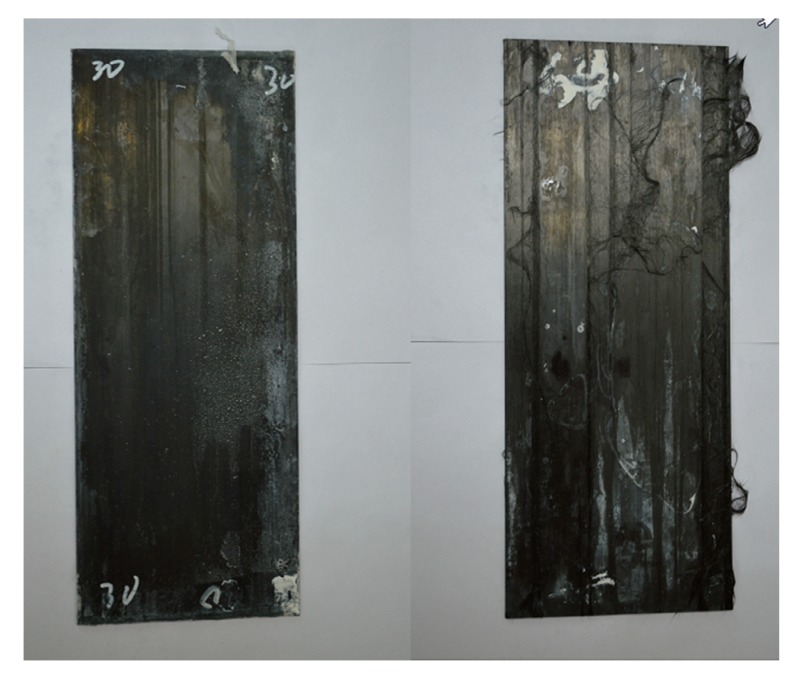
Coated specimen (left side) and naked specimen (right side) immersed in 60 °C alkaline solution for 135 days.

It is worth noting that specimen immersed in 60 °C alkaline solution for 135 days was seriously damaged, as shown in Figure 6 on the right compared with that coated by adhesive shown on the left. The behavior of naked CFRP plates immersed in water and alkaline solution has been investigated elsewhere [26,27]. The diffusion parameters of naked CFRP plates are shown in Table 2.

With regard to the water uptake behavior of coated specimens in Figure 5c,d, the water uptake behavior of adhesive coating was investigated elsewhere [28] where the specimen size is 25 mm × 25 mm × 3 mm and the water uptake parameters can be found in Ref. [28]: for T1 adhesive immersed in 20 °C water, M_∞T_ equals 2.82%, D equals 7.8 × 10^−6^ mm^2^/s; for T1 adhesive immersed in 50 °C water, M_∞T_ equals 2.52%, D equals 3.43 × 10^−5^ mm^2^/s.

**Figure 7:**
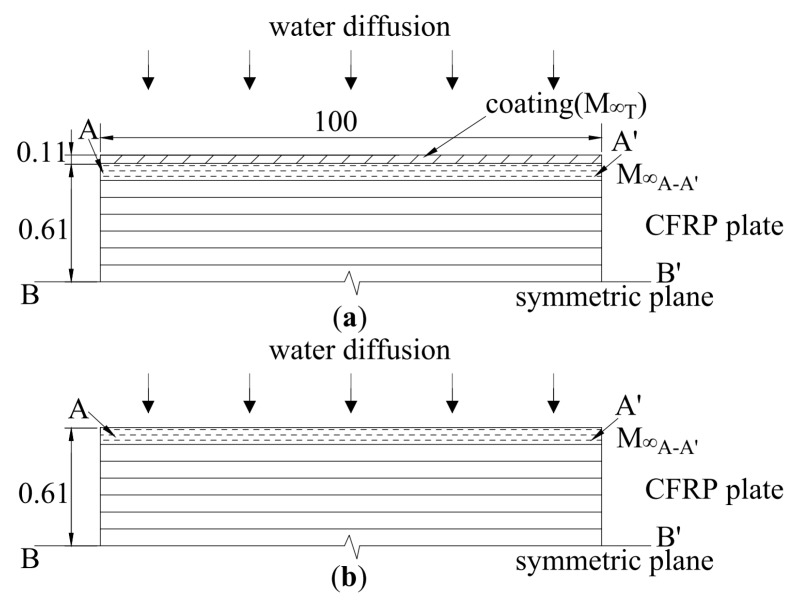
Cross-Section of (**a**) Coated CFRP plate; (**b**) Naked CFRP plate.

Consider the cross-section of the adhesive coated CFRP plate in Figure 7a and that of the naked CFRP plate shown in Figure 7b and B-B’ in Figure 7a,b is the symmetric plane. Both specimens are meshed into thin layers as shown. The fiber direction is perpendicular to the cross-section plane as shown in Figure 7a,b. Only the water diffusion perpendicular to the plate is considered and the diffusion direction is shown in Figure 7a,b as the length or width of the CFRP plate and the adhesive coating is an order of magnitude larger than their thickness.

**Figure 8:**
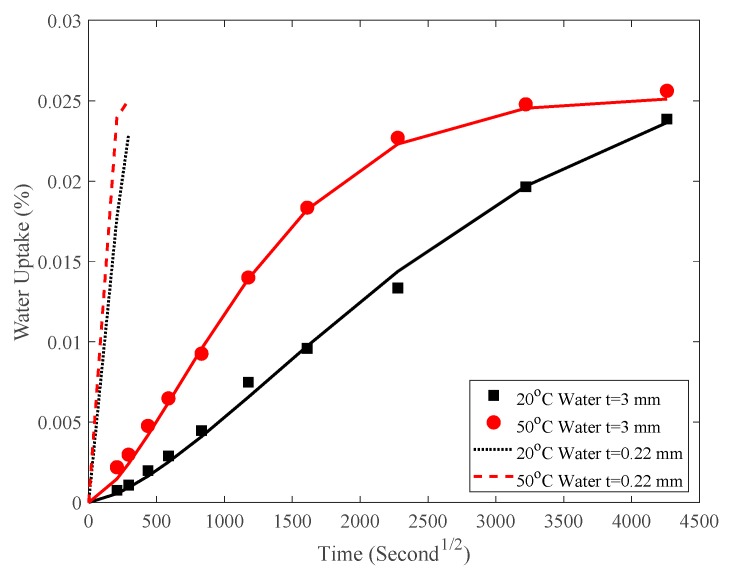
Water uptake of T1 adhesive.

The cross-section of the adhesive coating is shown in Figure 7a and the size of the adhesive coating is 250 mm × 100 mm × 0.11 mm. As an example, substituting an adhesive coating thickness *h* = 0.22 mm and diffusion parameters for T1 adhesive described previously into Equation (3) gives the water-uptake behavior of adhesive in the first diffusion stage as shown in Figure 8. The saturation time is much shorter compared with the first point at t = 778 S^1/2^ in Figure 5. Furthermore, the equilibrium amount of absorption M_∞T_ of T1 adhesive coating is much larger than that of CFRP plate M_∞_ shown in Table 2. Consequently it can be assumed that the adhesive coating reaches a saturation level in a much shorter time; the boundary condition of the coated CFRP plate in Figure 7a is the same as that of the naked CFRP plate shown in Figure 7b that is: the outer thin layer of the CFRP plates A-A’ as shown reaches its M_∞_ in a short enough time [21]. As a result, theoretically in the initial stage, the boundary conditions in Figure 7a,b are the same and subsequently the diffusion parameter *D* is the same too.

**Figure 9:**
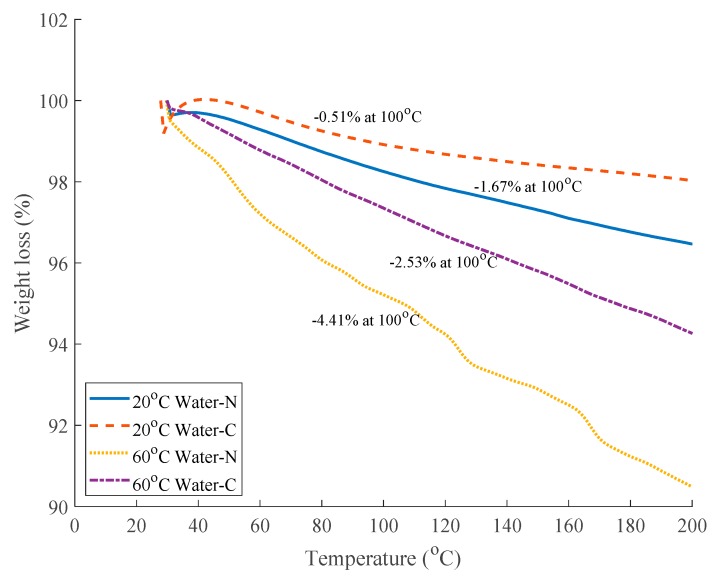
Thermogravimetric analysis of specimens immersed for 135 days.

However, this is not the case for the subsequent aging process. The result of thermogravimetric analysis of naked specimens and coated specimens is shown in Figure 9 and the weight loss at 100 °C is due to the water evaporation which equals the water uptake of these specimens. Although due to the non-uniform water distribution in the specimens the thermogravimetric analysis cannot give accurately average water uptake of the specimens, it can still reflect the water uptake of specimens qualitatively. A comparison of naked specimens and coated specimens immersed in 20 °C or 60 °C water for 135 days shows that the adhesive coating can significantly slow down the water diffusion. For instance, for specimens immersed in 60 °C water for 135 days, naked specimen and coated specimen have water uptakes of 4.41% and 2.53%, respectively. As explained previously, the two cases in Figure 7a,b have the same diffusion parameter *D* in the initial stage. However, as shown in Figure 9, the two cases have different water uptakes after 135-day immersion. Hence, it can be concluded that the water diffusion parameter *D* may increase during immersion due to the gradual degradation of the interfacial bonding between fiber and resin matrix and hence *D* is not a constant value. The adhesive coating can significantly slow the degradation of the interfacial bonding between fiber and resin matrix and subsequently the water diffusion in specimens.

### 3.2. DMTA Analysis

**Figure 10:**
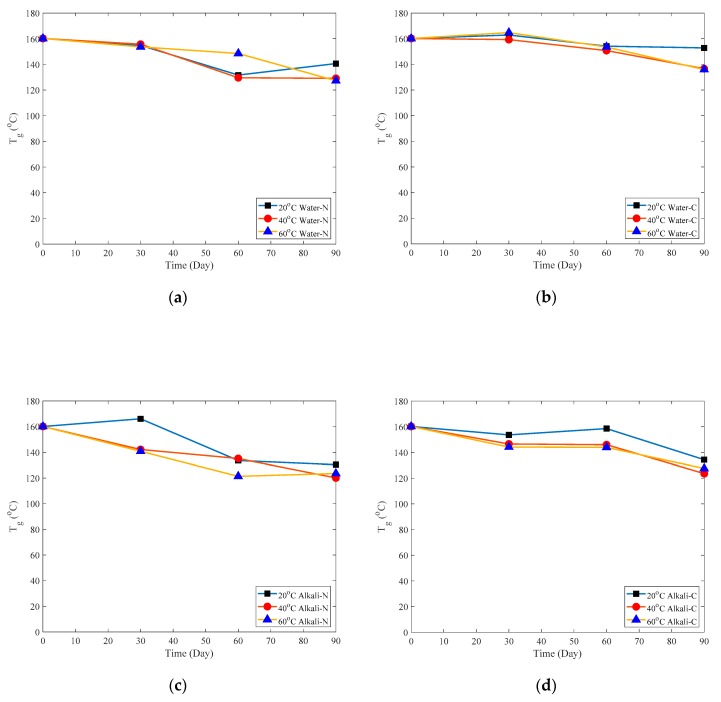
Variation of T_g_ for: (**a**) Naked plate immersed in water; (**b**) Naked plate immersed in Alkaline solution; (**c**) Coated plate immersed in water; (**d**) Coated plate immersed in Alkaline solution.

Figure 10 shows that the glass transition temperature decreases during the three-month immersion for both naked CFRP plates and adhesive-coated CFRP plates. After a three-month immersion, the glass transition temperatures of specimens reduce slightly by 19.7% and 15.6% for naked specimens and adhesive coated specimens, respectively.

**Figure 11:**
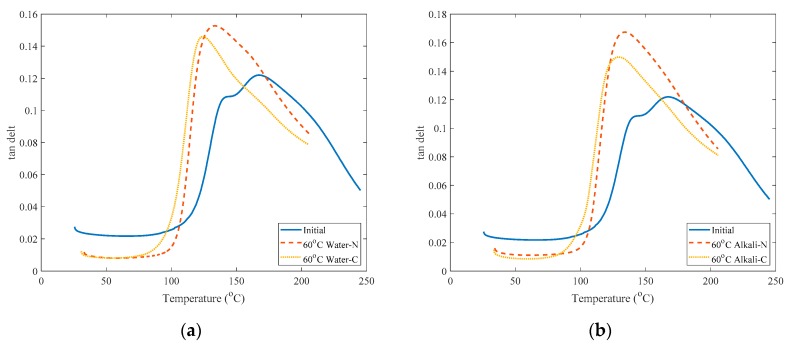
Comparison of tan δ between initial specimens and those immersed for three months.

The curves of damping factor tan δ for the initial specimen, naked specimen and adhesive-coated samples immersed in water or alkaline solution for three months are shown in Figure 11. The peak value tan δ_max_ of tan δ reflects the bonding performance of the interface between resin and fiber and the lower value of tan δ_max_, the better interfacial bonding strength is [11]. Hence, the ratio of initial-specimen tan δ_max0_ over δ_max_ of specimen aged for three months shown in Table 3 reflects the degradation of interfacial bonding strength. Higher ratio means more degradation of the interfacial bonding properties. It shows that for adhesive coated specimens immersed in 60 °C water for three months, tan δ_max_/tan δ_0max_ equals 1.20 which is slightly lower than the naked specimens in the same aging condition that is 1.25.

However, for adhesive coated specimens immersed in 60 °C alkaline solution for three months, tan δ_max_/tan δ_0max_ equals 1.23 which is much smaller than that of naked specimens in the same aging condition that is 1.37. This means the adhesive coating has a positive influence on the interfacial bonding strength of immersed specimens especially those immersed in the alkaline solution. 

However, from Figure 11 and Table 3, the interfacial bonding between fiber and resin matrix of adhesive-coated samples immersed in water or alkaline solution for three months is still considerably worse than that of the initial specimen.

### 3.3. Tensile Properties

**Figure 12:**
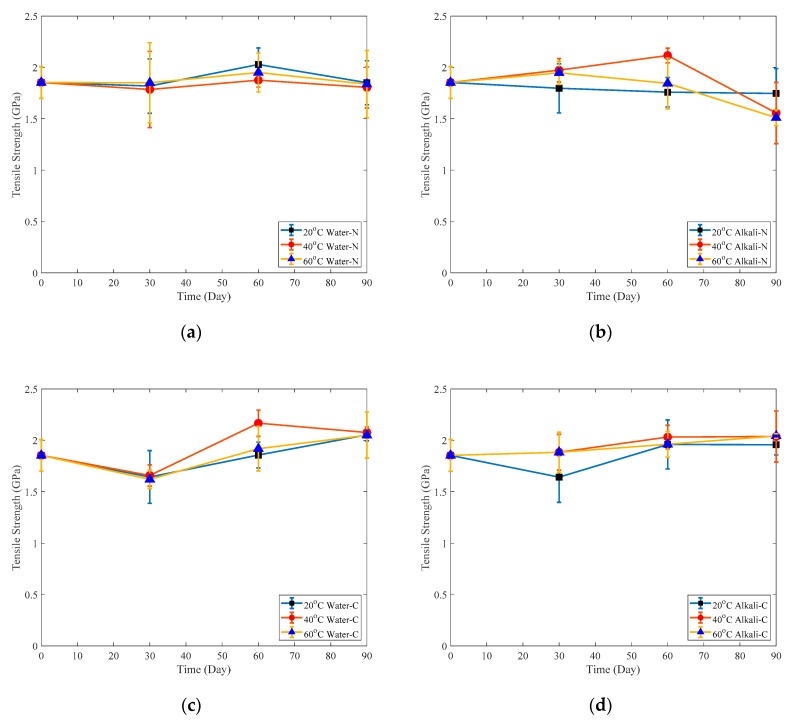
Tensile strength of: (**a**) Naked plate immersed in water; (**b**) Naked plate immersed in alkaline solution; (**c**) Coated plate immersed in water; (**d**) Coated plate immersed in alkaline solution.

**Figure 13:**
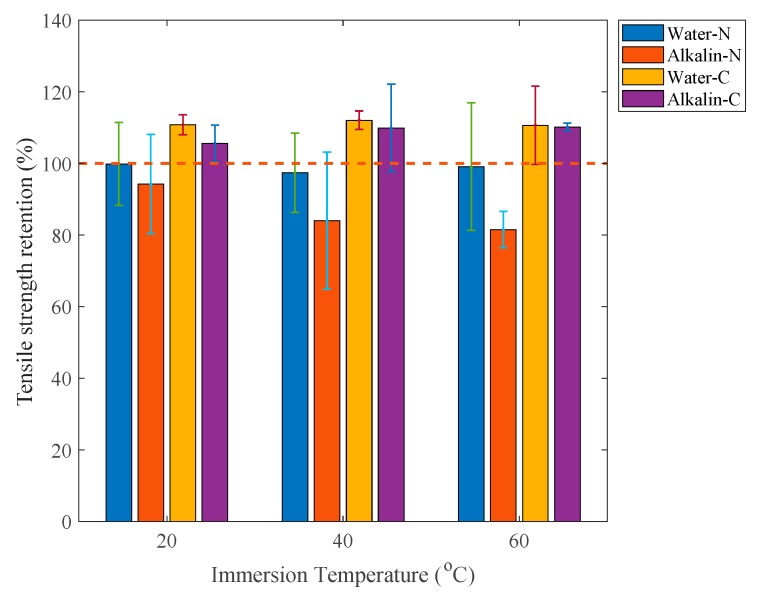
Tensile strength retention of specimens immersed for three months.

The tensile properties of CFRP naked and adhesive coated plates immersed in the water are plotted as a function of time as shown in Figure 12a,c, respectively and those immersed in the alkaline solution are shown in Figure 12b,d. Corresponding tensile strength retentions of samples immersed for three months are shown in Figure 13. It shows that the tensile strengths of both naked and adhesive coated specimens immersed in water as well as adhesive-coated samples immersed in alkaline solution shown in Figure 12a,c,d, respectively, do not change apparently. By contrast, the tensile strength of naked samples immersed in alkaline solution degrade during three-month immersion as shown in Figure 12b, especially during the last immersion month. Tensile strength retentions of naked samples immersed in water after three-month exposure are closed to 100% as shown in Figure 13. However, the tensile strength retentions of naked samples immersed in 20 °C, 40 °C and 60 °C alkaline solution after three-month immersion are 94.2%, 84.0% and 81.5%, respectively. By contrast, the tensile strength of adhesive-coated samples immersed in water or alkaline solution increased by approximately 20% as shown in Figure 13. This is mainly due to the post-cure of the composite [29] and the protection from the adhesive coating.

**Figure 14:**
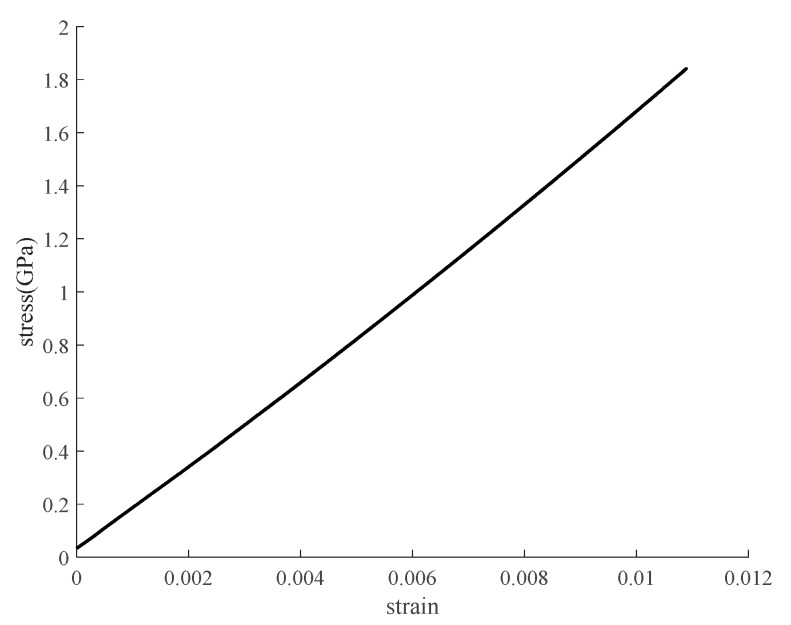
Tensile stress/strain relationship of naked CFRP plate immersed in alkaline solution for 90 days.

The tensile stress/strain relationship of a naked specimen immersed in alkaline solution for 90 days is shown in Figure 14. It is obvious that the CFRP plate still behaves elastically like unaged FRP although it is seriously damaged. The fracture strain of immersed specimens is shown in Table 4 and Table 5. It is obvious that the fracture strain of naked immersed specimens decreases during three months immersion and by contrast, that of coated immersed specimens increases slightly.

**Figure 15:**
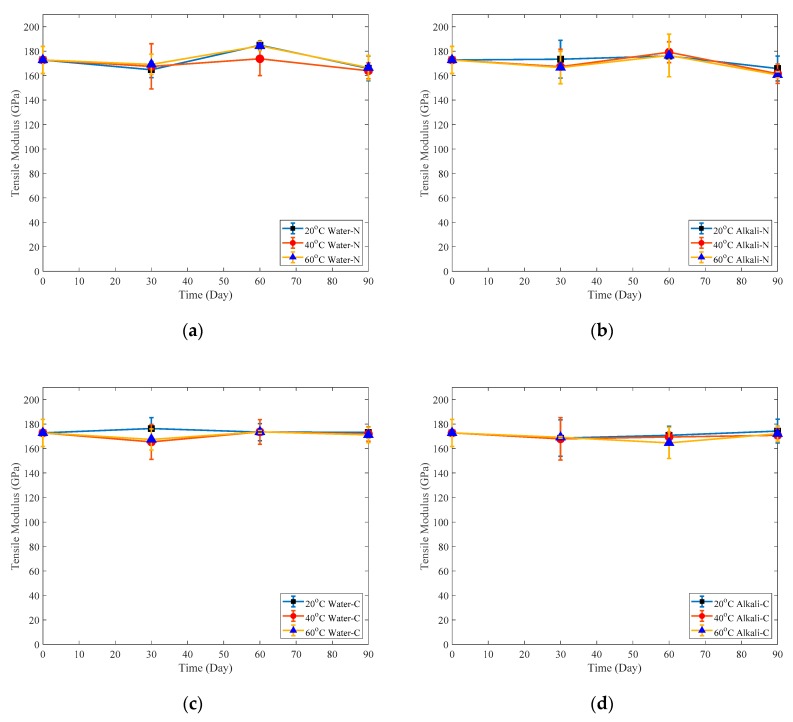
Tensile modulus of: (**a**) Naked plate immersed in water; (**b**) Naked plate immersed in alkaline solution; (**c**) Coated plate immersed in water; (**d**) Coated plate immersed in alkaline solution.

**Figure 16:**
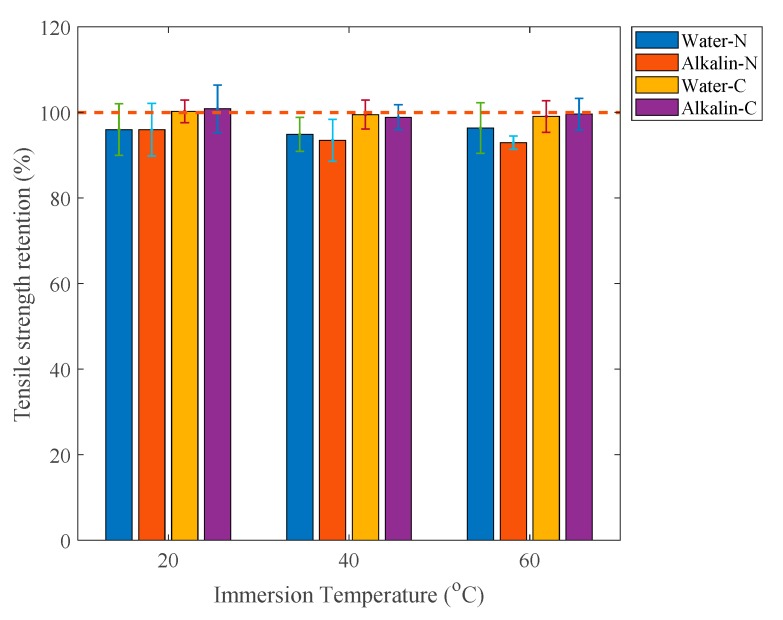
Tensile modulus retention of specimens immersed for three months.

The variation of specimen tensile modulus against time and the retention of tensile modulus of specimens immersed for three months are shown in Figure 15 and Figure 16, respectively. The immersion effect on tensile modulus of specimens is smaller than that on tensile strength. Figure 16 shows that after three-month immersion, the tensile modulus retentions of naked specimens are close to 95%. By contrast, those of adhesive-coated specimens are around 100%.

The tensile strength and modulus are determined by the fiber tensile properties and the interfacial bonding between fiber and resin matrix, which can be protected well by the adhesive coating from the analysis of tensile strength and modulus in this section. However, it has been described previously that the interfacial bonding between fiber and resin matrix degraded for coated specimens immersed for three months and it can be concluded that for these specimens, the degradation of the interfacial bonding between fiber and resin matrix is not serious enough to affect the tensile properties.

### 3.4. Chemical Analysis

It has been described previously that he naked CFRP plates immersed in 60 °C alkaline solution degrade more seriously in tensile strength compared with specimens in the other aging conditions which is also shown in Figure 6. The mechanism is explained in this section based on the FTIR tests which were conducted for the initial naked CFRP plate and that immersed in water or alkaline solution for 135 days.

**Figure 17:**
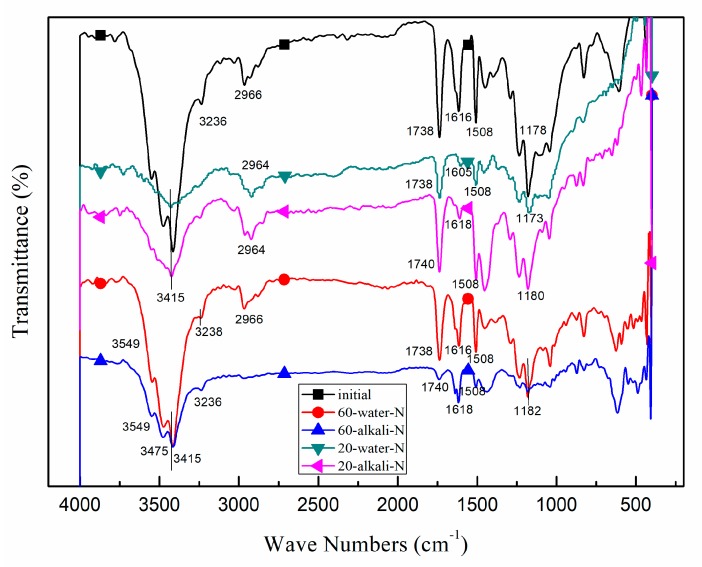
FTIR of the initial CFRP plate and that immersed for 135 days (numbers in the figure shows the specific value of the spectrum of absorption).

In Figure 17, the FTIR spectrum of naked samples immersed for 135 days is compared with that of the initial specimen. All these specimens have the absorption peak due to C=O group which means this group is stable during the immersion. However, it is found the naked specimens immersed in 60 °C alkaline solution for 135 days do not have absorption peak around 1180 cm^−1^, while those immersed in the other aging conditions have this peak. This change in absorption is because that the ester band(C–O) hydrolyzed for naked CFRP plates immersed in 60 °C alkaline solution [30].

### 3.5. In-Plane Shear Capacities

**Figure 18:**
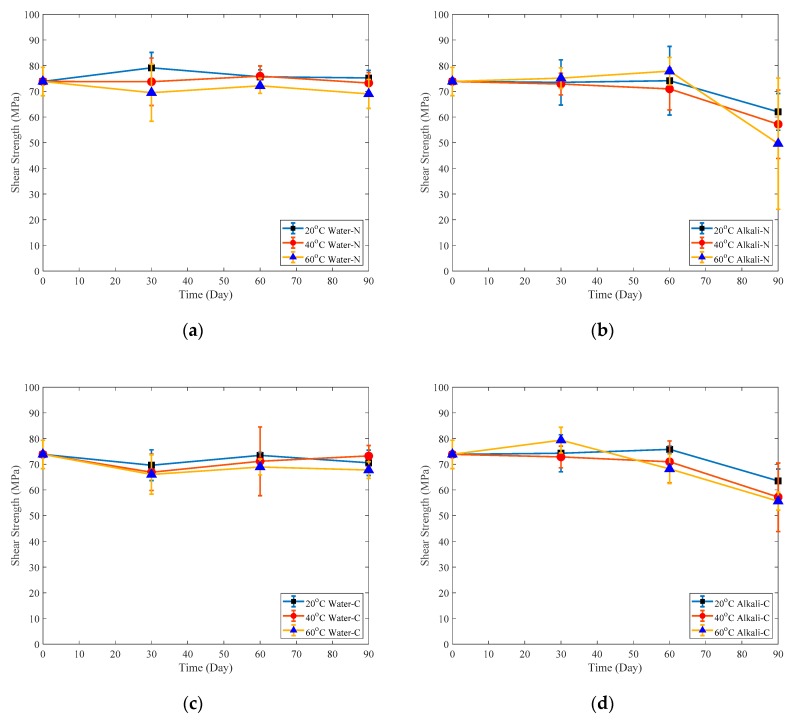
In-Plane shear strength of: (**a**) Naked plate immersed in water; (**b**) Naked plate immersed in alkaline solution; (**c**) Coated plate immersed in water; (**d**) Coated plate immersed in alkaline solution.

In-plane shear strengths of the naked CFRP plates and adhesive coated samples immersed in water or alkaline solution are plotted as a function of time as shown in Figure 18, which shows that the shear strength of samples immersed in water in Figure 18a,c almost does not degrade. However, the shear strength of samples immersed in alkaline solution decreases much faster than that immersed in water especially in the last immersion month. The average shear strength of samples immersed in the alkaline solution decrease by 13.4% for naked samples and 9.46% for adhesive coated samples in the last immersion month. Shear strengths of adhesive-coated samples immersed in alkaline solution for three months reduced by 14.0%, 22.6% and 24.7% for immersion temperatures of 20, 40 and 60 °C, respectively. These numbers for naked samples under the same aging conditions are 16.0%, 22.6% and 32.8%, respectively.

**Figure 19:**
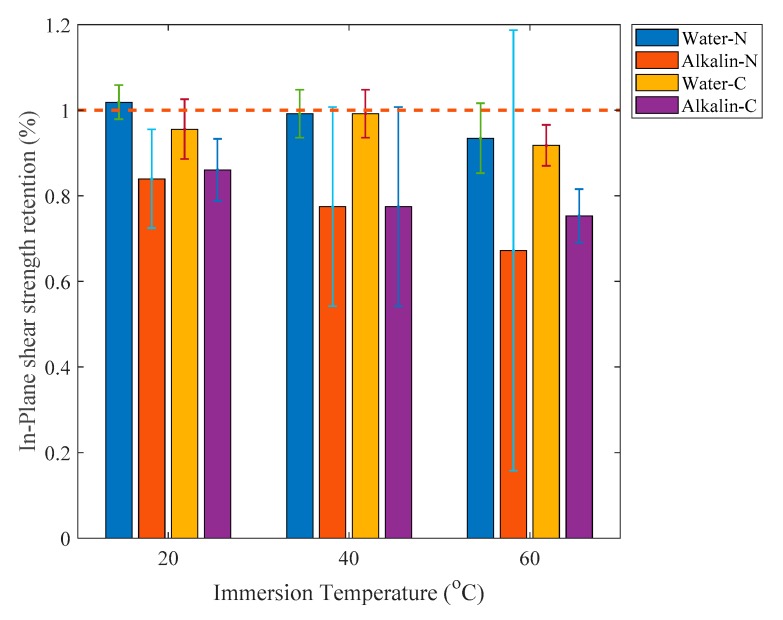
In-Plane shear strength retention of specimens immersed for three months.

The in-plane shear strength retention of samples immersed for three months is shown in Figure 19. If compared with the tensile strength data shown in Figure 13 it is obvious that the protective effect of the adhesive coating on shear strength of samples immersed for three months is smaller than that on tensile properties. The in-plane shear strength retention of CFRP plates reflects the properties of epoxy resin and the interfacial bonding between fiber and resin. It has been described previously that the interfacial bonding between fiber and resin matrix can be protected well by the adhesive coating. Hence, the in-plane shear strength reduction of adhesive-coated samples may be mainly due to the degradation of the epoxy resin. Furthermore, it may be concluded that the epoxy resin is more likely to be sensitive to temperature instead of water uptake because lower water uptake due to the adhesive-coating protection does not lead to obviously better properties of in-plane shear strength.

## 4. Conclusions

In this article, the aging of naked pultruded CFRP plates and adhesive coated pultruded CFRP plates immersed in water or alkaline solution is investigated. The following conclusions are given based on the current study:

(1) Water diffusion analysis such as Fick’s law, two-stage model and finite element analysis normally assume constant diffusion speed by assuming constant diffusion parameter. However, from the analysis in this study, the diffusion speed is slow at the beginning stage and may increase as the degradation of the interfacial properties between fiber and resin matrix.
(2) The adhesive coating can slow the degradation of the interfacial bonding between fiber and resin matrix and subsequently the water diffusion in the CFRP plates especially for samples immersed in 60 °C alkaline solution. As a result, the adhesive coating can significantly protect the tensile properties of CFRP plates immersed in high-temperature alkaline solution.
(3) The adhesive-coating protective effect on in-plane shear strength is smaller than that on tensile properties. It is also concluded that the adhesive coating does not have a significant protective effect on the epoxy resin of immersed samples. The epoxy resin may be more likely to be sensitive to temperature instead of water uptake.

## Figures and Tables

**Table 1:** Components of alkaline solution.

Component	Ca(OH)_2_	NaOH	KOH
Content (g/L)	118.5	0.9	4.2

**Table 2:** Diffusion parameters of naked CFFR plates.

Characteristic	20 °C Water-N	40 °C Water-N	60 °C Water-N	20 °C Alkaline-N	40 °C Alkaline-N
$M_{\infty }$	0.106%	0.140%	0.198%	0.176%	0.192%
D(× 10^−7^ mm^2^/s)	1.75	2.50	37.8	2.23	2.83
k(× 10^−5^/s^1/2^)	72.0	75.9	77.4	48.8	53

**Table 3:** Tan δ variation of specimens immersed for three months.

Characteristic	Initial	60 °C Water-N	60 °C Water-C	60 °C Alkaline-N	60 °C Alkaline-C
tan δ_max_	0.122	0.153	0.146	0.167	0.150
tan δ_max_/tan δ_0max_	1	1.25	1.20	1.37	1.23

**Table 4:** Fracture strain of naked immersed specimens.

Fracture Strain	20 °C Water-N	40 °C Water-N	60 °C Water-N	20 °C Alkaline-N	40 °C Alkaline-N	60 °C Alkaline-N
Fracture strain at 0 day	0.0107	0.0107	0.0107	0.0107	0.0107	0.0107
Fracture strain at 30 days	0.0110	0.0107	0.0109	0.0104	0.0118	0.0117
Fracture strain at 60 days	0.0110	0.0108	0.0106	0.0100	0.0118	0.0105
Fracture strain at 90 days	0.0112	0.0110	0.0110	0.0105	0.0096	0.0094

**Table 5:** Fracture strain of coated immersed specimens.

Fracture Strain	20 °C Water-C	40 °C Water-C	60 °C Water-C	20 °C Alkaline-C	40 °C Alkaline-C	60 °C Alkaline-C
Fracture strain at 0 day	0.0107	0.0107	0.0107	0.0107	0.0107	0.0107
Fracture strain at 30 days	0.0093	0.0100	0.0097	0.0097	0.0112	0.0111
Fracture strain at 60 days	0.0107	0.0125	0.0111	0.0115	0.0120	0.0119
Fracture strain at 90 days	0.0119	0.0121	0.0120	0.0112	0.0119	0.0119
